# An identity-based learning community intervention enhances the lived experience and success of first-generation college students in the biological sciences

**DOI:** 10.1038/s41598-024-60650-1

**Published:** 2024-05-03

**Authors:** Deborah J. Wu, Tracie M. Gibson, Linda M. Ziegenbein, Randall W. Phillis, Caralyn B. Zehnder, Elizabeth A. Connor, Nilanjana Dasgupta

**Affiliations:** 1 Department of Psychology, Stonehill College, Easton, USA; 2 Office of Student Success and Diversity, College of Natural Sciences, University of Massachusetts, Amherst, USA; 3 Department of Biology, University of Massachusetts, Amherst, USA; 4 Department of Psychological and Brain Sciences, University of Massachusetts, Amherst, USA

**Keywords:** Human behaviour, Psychology

## Abstract

Working-class first-generation (FG) college students are underrepresented in higher education and STEM. Using a longitudinal quasi-experiment, we tested the impacts of a living learning community (LLC) in the biological sciences on FG students in their first year of college (Semester 1: *N* = 243; Semester 2: *N* = 199), across three cohorts (2018–2019, 2019–2020 and 2020–2021). Participation in the LLC enhanced FG students’ belonging, confidence, motivation, grades, knowledge of the social relevance of biology, and reduced STEM anxiety compared to a control group of FG students not in an LLC. LLC participation also increased retention in biological science majors one-year post-intervention compared to the control FG group. Moreover, LLC participation closed the academic gap between FG students in the LLC and honors students from college-educated families in a separate honors LLC. Benefits of the LLC intervention remained stable despite the COVID-19 pandemic, when living together became impossible, producing positive effects across cohorts from pre-pandemic to in-pandemic. Our results suggest that affinity-based learning communities—with or without shared housing—in the transition to college enhance academic thriving, persistence, and reduce social class driven achievement gaps in STEM.

## Introduction

The demographics of scientists, engineers, and technologists in the United States do not represent the American population at large. Significantly fewer people from working-class backgrounds, Black and African Americans, Hispanics, and women occupy STEM higher education and professions^1^, failing to live up to the nation’s promise of equal opportunity and limiting scientific innovation that comes from diverse minds working together^2,3^. Furthermore, onramps for young people to enter and persist in STEM pathways are few^4^. Consider the case of working-class first-generation (FG) students who are the first in their family to pursue 4-year college degrees. Although college degrees offer the most reliable path to social mobility in the United States, FG students face multiple barriers^5–7^. They are less likely to enter and complete college because of financial costs, family responsibilities, and limited social networks providing contacts within academia^8,9^. They are also less likely to major in STEM in college, compared to peers with college-educated parents who are often called continuing-generation (CG) students^8^. At each step in the STEM pathway, fewer FG students receive college degrees and other STEM credentials necessary to pursue STEM careers and social mobility^8–11^.

Our research focuses on three key barriers that prevent working-class FG students from graduating college with STEM degrees: (1) the individualistic culture of universities that expects students to be autonomous and find their own path^12^, (2) disconnection from resource-rich social and professional networks that open doors to academic opportunities^13^, and (3) lack of authentic relationships with academic role models^5^. The culture at many universities emphasizes independent self-directed learning, creating a cultural mismatch to working-class norms that often emphasize interdependence^11,12^. Previous research has shown that increased perception of cultural mismatch in college is associated with greater stress and lower academic performance among FG students^12,14^. Compared to CG students, FG students are also less likely to receive college-related advice from their parents^5,8^ and are more likely to attend under-resourced high schools^15^, often resulting in less preparation for college and limited academic networks that are gateways to internship, college, and career opportunities. Finally, FG students often do not have academic role models from similar backgrounds with whom they can develop authentic relationships and receive instrumental guidance^16,17^. We designed and implemented a theory-driven intervention targeting these three barriers and tested its impact on FG students’ success in the biological sciences.

Surprisingly few studies have rigorously tested evidence-based interventions targeting FG student success in STEM. A few notable exceptions are studies using values-oriented interventions to reduce achievement gaps between FG and CG students^18–21^. These studies showed that when working-class FG students reflected on important personal values (value-affirmation) or described how their course content in biology was meaningful to them (utility-value induction), their subsequent grades in introductory biology were significantly higher, and they were more likely to persist in biology the following semester, compared to FG students in a control condition^18,19,21^. Value-affirmation also improved FG students’ performance on a math test in a laboratory situation^20^. These studies are important because they show that brief interventions benefit FG students in STEM; yet, like all studies they also have limitations. First, by focusing solely on students’ mental appraisals of STEM, the implicit onus is on students who are marginalized in STEM to adjust their mindset to fit within the existing academic system rather than changing institutional learning environments to meet the needs of marginalized students. Second, the primary student outcomes in these past studies were grades and retention in academic majors, not lived experiences in STEM learning environments. Lived experiences such as belonging in STEM, confidence, motivation, and anxiety were either unexamined or remained unchanged by the intervention^18,19,21^. The present research addresses these limitations by designing and field-testing a STEM living-learning environment on students’ lived experiences, performance, and retention in biology one-year post-intervention.

Our theoretical framework is the Stereotype Inoculation Model^22^ that has empirically demonstrated that in achievement-oriented contexts where individuals face stereotypes and discrimination, exposure to ingroup members protects their feelings of belonging, confidence, motivation to persist, and future aspirations^23^. Informed by this model and the barriers that working-class FG students face in college and STEM, our intervention created a different type of learning environment for undergraduate biology by introducing three important changes. First, we created a living-learning community (LLC) of students who were the first in their family to attend a 4-year college and were interested in the biological sciences. Students lived together as a cohort, took two classes together, providing multiple opportunities to form a close-knit affinity group. Second, we brokered meaningful relationships with faculty instructors, academic advisors, and visiting scientists to grow students’ academic and professional network. Third, we provided access to peer mentors who helped students develop concrete steppingstones for the next steps of their academic journey. The abrupt advent of the COVID-19 pandemic introduced an unexpected opportunity to separately examine the impact of the intervention when students lived together and learned together (pre-pandemic) versus when they learned together in a cohort without living together (in-pandemic).

Our work expands upon prior research on LLCs and summer bridge programs that have yielded mixed results for underrepresented students^24–26^. LLCs gained popularity because participating students report greater belonging and social connection compared to non-participating students^27–29^. However, most LLC participants in past research have been White, middle- and upper-middle class students from college-educated families^25,30^. When underrepresented students participated in LLCs, they continued to be in the numeric minority within the LLC^26,29–31^, which is likely responsible for the mixed results. While some studies found that women, racial minorities, and FG students benefited from LLCs^29,32–34^, others found that racial ethnic minority students reported lower feelings of belonging compared to White students^24–26^.

Most past research did not recruit students into LLCs based on shared identities (race, gender, social class, etc.) for students interested in STEM, except for a few notable studies. In one study, women in an all-female LLC in STEM were more likely to graduate with a STEM degree^35^, and an LLC designed for low-income students in STEM showed benefits for students’ first-year retention^36^. In another study, Black students in an LLC specifically for racial minority students in STEM earned Bachelor’s and doctorate degrees in STEM at higher rates than Black students who were not in this LLC^37,38^. In all these studies, the outcomes narrowly focused on degrees granted and/or retention. Our interest was broader—while we examined grades and retention, we also measured students’ psychological lived experiences of belonging, confidence, anxiety, and motivation—variables that we predict are early indicators of future performance, persistence, or attrition^8^.

Summer bridge programs, that are immersive on-campus experiences designed to orient students before the first year of college, are often directed at underrepresented minority students. These have yielded benefits. Specifically, Black, Hispanic, and Indigenous students who attend STEM summer bridge programs were more likely to graduate college than matched control groups^39^. However, this research and others that measured the quantitative impacts of LLCs in STEM have largely focused on retention in STEM majors as the primary outcome^40,41^. We expand upon prior research by assessing FG students’ lived experiences in academic STEM settings over time with the hypothesis that lived experiences are precursors of persistence and retention in STEM over time. Furthermore, we focus specifically on the impacts of a learning community program for working-class FG students in STEM, a population that, surprisingly, has not been the focus of any cohort-based intervention program in STEM. To address these gaps, we conducted a longitudinal quasi-experiment to test the impact of a biological science focused LLC on FG students, over two semesters. Semester 1 followed students through their first semester in college while they took their first introductory biology class. Semester 2 followed them through their second semester in college while they took a second required biology class. FG students in the LLC, our intervention group, were compared to two groups: (1) FG students with biological science interests who were not in the LLC, and (2) a group of primarily CG students with biological science interests, who were in a different honors college LLC.

Undergraduate students were recruited in the summer before their first year in college. Students from working-class families who were first in their families to attend college and who had expressed interest in the biological sciences (i.e., biology, microbiology, biochemistry and molecular biology, neuroscience, animal sciences, or veterinary science) were invited to apply for a living-learning community for FG students. Three cohorts of students were recruited in academic years 2018–2019, 2019–2020, and 2020–2021. Each year, among those who applied to the LLC, 19–38 FG students were quasi-randomly assigned to the LLC (intervention group) or no intervention (control group) with one caveat: the LLC oversampled students from groups that are historically underserved in STEM. Black and Hispanic students and Pell grant recipients (students whose family income was below the federal poverty level) were oversampled into the LLC as compared to the control group. For the 2018–2019 and 2019–2020 cohorts, the control group comprised FG students who had applied to the LLC but were randomly assigned to the control group plus other FG students in the same first-year class who were interested in biology but had not applied to the LLC. During the unprecedent stress of the COVID pandemic, we elected to admit most of the applicants into the intervention group for our last cohort (2020–2021; 38 out of 44 applicants, or 86%). Demographics of students who were invited to apply, who were admitted (vs. not admitted) into the program, and who joined (vs. did not join) the program are presented in Supplementary Tables S1 and S2.

Several key differences characterized the college experiences of FG students in the LLC as compared to the control group. The LLC was intentionally designed to foster academic and social interdependence. Students took introductory biology and a first-year seminar together as a cohort, lived on the same floor of a residence hall, and participated in group-based social activities once a semester. The LLC increased access to academic and professional networks through biology guest speakers, who came to FG students’ first-year seminars to present their research, and through FG peer mentors who were juniors or seniors in biological science majors. In contrast, FG students in the control group experienced the typical individualistic learning environment, taking introductory biology in a large lecture-based class with mostly CG students. FG students in the control group lived in the same residence halls as LLC participants, but they were not grouped with FG peers. They took a first-year seminar, but they were not grouped with FG students and the content was not connected to biology. However, there were also important similarities between the two groups. In each semester, both FG LLC and FG control group students took introductory biology with the same professor who taught both sections of the course in an identical manner—using the same syllabus, lectures, exams, homework, and grading system. We only recruited control students who were in the biology section taught by the same professor who also taught the FG LLC students, to better match the biology content taught in class, teaching style, and performance evaluation metrics across the two conditions.

In addition to comparing FG students in the LLC with FG students in the control group, we included a second comparison group—honors college students participating in an honors biology LLC. Honors college students make up approximately 13% of each incoming first-year class at this university and are admitted based on high achievement in high school. Upon college admission, honors students interested in the biological sciences were invited to apply to a biology-focused LLC housed within the honors college. The vast majority of students in the honors LLC came from middle- and upper-middle class college-educated families (88–90%). Like the FG biology LLC, students in the honors LLC took introductory biology and a first-year seminar as a cohort, lived together in a common residence hall, and had biological science professors visit their seminar as guest speakers who presented their research and provided research opportunities. While the content of the honors version of introductory biology was similar to the class FG students were taking, the teaching format was different (team-based learning vs. lecture-style teaching) and the instructors who taught introductory biology for the honors LLC were different from those who taught students in the FG LLC and the control conditions.

As noted earlier, the residential component of the LLC was suspended during the COVID-19 pandemic, which introduced complications but also an unexpected opportunity to compare the joint impact of living-plus-learning together (pre-pandemic) versus the isolated impact of learning together without living together (in-pandemic). Specifically, during the spring semester of 2020, in-person learning and residential living was disrupted midway through the semester, which continued through the 2020–2021 academic year when all classes were virtual and residential living for the LLCs was suspended. Due to the timing of these disruptions, by aggregating Semester 1 cohorts across 3 years, we were able to compare pre-pandemic cohorts who experienced living plus in-person learning together versus an in-pandemic cohort who experienced virtual learning together without the shared living component. Unfortunately, as residential life and virtual learning for Semester 2 cohorts was disrupted during the spring 2020 cohort, we were unable to separately examine pre-pandemic vs. in-pandemic effects in Semester 2.

Our outcome measures included students’ psychological experiences in biology (feelings of belonging in biology, motivation to persist, confidence, and anxiety about the course), understanding the social relevance of biology, final grades in biology, and retention in biological science majors one year after the LLC had concluded. Participants were surveyed twice, in the middle and end of each semester, to assess the consistency of their responses. Three research questions guided our study:

1. Does participation in a biology LLC enhance first-generation students’ academic experiences in biology (belonging, anxiety, motivation, and confidence), increase their understanding of the social relevance of biological research, and boost final grades as compared to the control group of FG students not in an LLC?
2. Does participation in a biology LLC increase FG students’ persistence in biological science majors one-year post-intervention compared to the control group of FG students not in the LLC?
3. Does participation in an LLC close gaps between first-generation students and honors college students in terms of academic experiences in biology and retention in biological science majors?

## Results

### Manipulation check

Because student assignment to condition was quasi-random as Black, Hispanic, and Pell grant recipient students were oversampled into the FG LLC, we conducted a manipulation check to compare self-reported college preparation for participants in the FG LLC group compared to the FG control group (see Table S2 for the demographic composition of students in the FG LLC and the FG control group). Specifically, we asked participants three manipulation check questions during participants’ first survey on 7-point scales ranging from “not at all” (1) to “very much” (7): (1) how much their family had prepared them for college, (2) how much their high school had prepared them for college-level biology, and (3) to what extent had they assumed, while in high school, that they were college bound. We conducted independent samples *t*-tests comparing the FG LLC to the FG control condition. Compared to the FG control condition (*M* = 5.30, *SD* = 1.67), those in the FG LLC reported less guidance from their family to prepare them for college (*M* = 4.38, *SD* = 1.88; *t*(147) = 3.09, *p* = 0.002, *d* = 0.51). No significant differences emerged in high school preparation (FG control: *M* = 4.60, *SD* = 1.65; FG LLC: *M* = 4.63, *SD* = 1.70; *p* = 0.929) or in college-bound expectations (FG control: *M* = 6.87, *SD* = 0.38; FG LLC: *M* = 6.71, *SD* = 0.81; *p* = 0.099) between the FG LLC versus FG control condition.

As a secondary check, we compared FG LLC to the honors LLC condition, finding that for all questions, FG LLC students felt less prepared for college than the honors college group (*p*s ≤ 0.015, *d*s ≥ 0.37). These results confirmed that: (1) those in our FG LLC group were similar to the FG control group in terms of high school preparation but more disadvantaged in terms of access to college guidance from family; and (2) students in the honors LLC who were mostly from college-educated families felt more prepared for college than FG LLC students. In addition to these manipulation checks, to ensure that the effect of the intervention on student outcomes was not biased by demographic differences between conditions, we statistically controlled for student race, gender, and SAT scores using Analyses of Covariance (ANCOVA). Results showed that the effect of the FG LLC intervention on student outcomes remained statistically significant even after controlling for demographics (see Supplementary Materials for details).

### Data analytic strategy

For Semester 1, we compared the effect of the intervention on student outcomes pre-pandemic vs. in-pandemic across two time points by conducting a series of mixed model Analysis of Variance (ANOVA) tests with Condition (control, FG LLC, honors LLC), Time (middle vs. end of semester), and Cohort (pre-pandemic, in-pandemic) as independent variables for each of the dependent measures. For Semester 2, we conducted similar ANOVAs as Semester 1, except that Cohort (pre-pandemic, in-pandemic) was not used as an independent variable because the timing of the pandemic in relation to our study prevented clean pre- and in-pandemic sub-samples.

If an omnibus significant effect emerged on any ANOVA, follow-up post hoc tests were conducted using Bonferroni corrections to adjust for multiple comparisons. Sensitivity power analyses (GPower 3.1^42^) revealed 95% power for an effect size of *d* = 0.19 in Semester 1 (243 participants; 6 groups, 2 measurements across time) and *d* = 0.24 in Semester 2 (199 participants; 3 groups, 2 measurements across time).

### Semester 1 Results

#### Belonging

**Figure 1:**
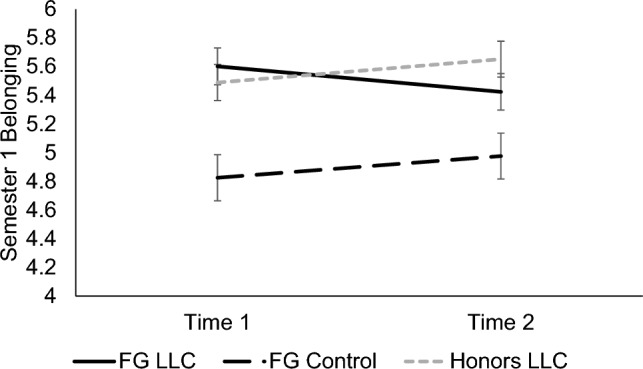
Participants’ feelings of belonging in biology in Semester 1. Means of participants’ feelings of belonging in their introductory biology course in Semester 1 for each timepoint are shown, separated by condition. Error bars signify ± 1 standard error.

A significant main effect of condition, *F*(2, 237) = 10.27, *p* < 0.001, *d* = 0.58, revealed that across the semester, FG students in the LLC felt a stronger sense of belonging in biology (*M* = 5.49, *SE* = 0.12) than FG students in the control group (*M* = 4.71, *SE* = 0.14, *p* < 0.001, *d* = 0.38). FG LLC students were statistically similar to honors LLC students in belonging (*M* = 5.44, *SE* = 0.11, *p* > 0.99), but FG controls felt significantly less belonging in biology compared to the honors LLC (*p* < 0.001, *d* = 0.36). Additionally, a significant main effect of cohort, *F*(1, 237) = 20.26, *p* < 0.001, *d* = 0.58, revealed that students in pre-pandemic cohorts felt more belonging in biology (*M* = 5.54, *SE* = 0.08) than students in the pandemic cohort (*M* = 4.88, *SE* = 0.12). We found no significant effects of time, or interaction effects between time, condition, and cohort, *p*s ≥ 0.119). Means for each timepoint are graphed in Fig. 1.

#### Anxiety

**Figure 2:**
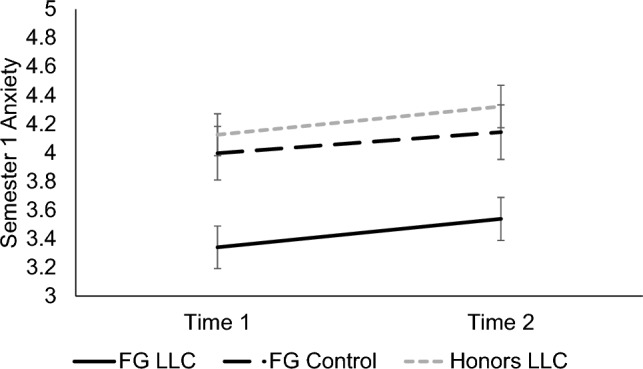
Participants’ feelings of anxiety in biology in Semester 1. Means of participants’ feelings of anxiety in their introductory biology course in Semester 1 for each timepoint are shown, separated by condition. Error bars signify ± 1 standard error.

A significant main effect of condition, *F*(2, 237) = 10.51, *p* < 0.001, *d* = 0.59, revealed that FG students in the LLC reported less anxiety about biology class (*M* = 3.42, *SE* = 0.13) than FG students in the control group (*M* = 4.10, *SE* = 0.17, *p* = 0.005, *d* = 0.29) and students in the honors LLC (*M* = 4.24, *SE* = 0.13, *p* < 0.001, *d* = 0.39). There was no difference in anxiety between the FG control vs. honors LLC conditions (*p* > 0.99). Additionally, a significant main effect of time, *F*(1, 237) = 5.02, *p* = 0.009, *d* = 0.34, showed an overall increase in anxiety across the semester from Time 1 (*M* = 3.81, *SE* = 0.09) to Time 2 (*M* = 4.03, *SE* = 0.10), for all conditions. No significant main effect emerged for cohort (pre-pandemic vs. in-pandemic); nor any interaction effects with time and condition, *p*s ≥ 0.191. See Fig. 2.

#### Motivation

**Figure 3:**
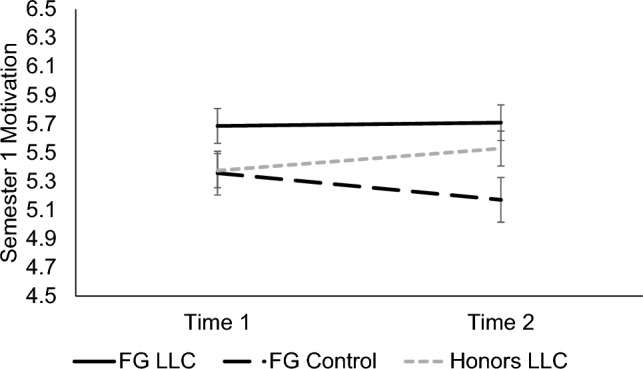
Participants’ feelings of motivation in biology in Semester 1. Means of participants’ feelings of motivation in their introductory biology course in Semester 1 for each timepoint are shown, separated by condition. Error bars signify ± 1 standard error.

We found a significant main effect of condition for motivation, *F*(2, 237) = 3.94, *p* = 0.021, *d* = 0.36, such that FG LLC students reported significantly more motivation in biology (*M* = 5.69, *SE* = 0.11) than FG control group students (*M* = 5.19, *SE* = 0.14, *p* = 0.019, *d* = 0.25). The honors LLC group did not significantly differ from either FG group (*p*s ≥ 0.291). A significant interaction of time x cohort, *F* (1, 237) = 5.74, *p* = 0.017, *d* = 0.31, indicated that during the pandemic students’ motivation decreased from Time 1 (*M* = 5.45, *SE* = 0.13) to Time 2 (*M* = 5.25, *SE* = 0.13, *p* = 0.038, *d* = 0.27), whereas before the pandemic, their motivation did not change significantly over time, *p* = 0.234. There were no other significant effects, *p*s ≥ 0.076. See Fig. 3.

#### Confidence

Students’ confidence in biology did not vary significantly by condition or by time, *p*s ≥ 0.080. A significant time x condition interaction, *F*(2, 238) = 4.43, *p* = 0.013, *d* = 0.38, indicated that in the FG control group students’ confidence decreased over time (*p* = 0.045, *d* = 0.26), whereas in the honors LLC condition students’ confidence increased over time (*p* = 0.017, *d* = 0.31). In the FG LLC, students’ confidence did not change significantly over time (*p* = 0.769). Moreover, a significant main effect of cohort, *F*(1, 235) = 4.11, *p* = 0.044, *d* = 0.26, indicated that students in pre-pandemic cohorts reported feeling more confident (*M* = 5.11, *SE* = 0.08) than those in the pandemic cohort (*M* = 4.82, *SE* = 0.12). No other significant effects were found, *p*s ≥ 0.053.

#### Understanding the relevance of biology in the real world

There were no significant effects of participants’ perceptions of the relevance of biology, *p*s ≥ 0.107.

#### Fall semester grades

**Figure 4:**
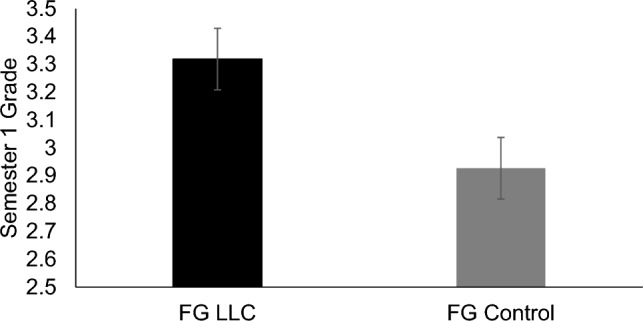
Participants’ grades in biology in Semester 1. Means of participants’ final grades in their introductory biology course in Semester 1 for each timepoint are shown, separated by condition. Error bars signify ± 1 standard error.

We tested whether students’ final grades in biology varied by condition. As a reminder, FG students in both LLC and control conditions took the same course with the same instructor (i.e., same content, teaching style, assignments, exams); the only difference was class size and the identity of fellow students—a class of FG peers only (LLC) vs. mostly CG peers (control condition). We did not compare grades between the FG LLC and the honors LLC because of differences in teaching style (i.e., team-based learning course vs. lecture-style course). As predicted, FG LLC students earned significantly higher grades in biology (*M* = 3.32 or B+, *SD* = 0.63) than FG control group students (*M* = 2.93 or between B and B−, *SD* = 0.67), *t*(134) = 3.56, *p* = 0.001, *d* = 0.61. In addition to the above-mentioned primary analysis of student grades, we also conducted a supplementary analysis of grades pre-pandemic vs. in-pandemic in response to the intervention using a 2 Condition (control vs. FG LLC) × 2 Cohort (pre-pandemic, in-pandemic) ANOVA. However, because students were allowed to take classes pass/fail during the pandemic, there were fewer grades during the pandemic cohort (*N* = 10 in control condition, *N* = 23 in FG LLC condition). Thus, while we report these results in the Supplementary Materials, we interpret them with caution. Values are graphed in Fig. 4.

### Semester 2 Results

#### Belonging

**Figure 5:**
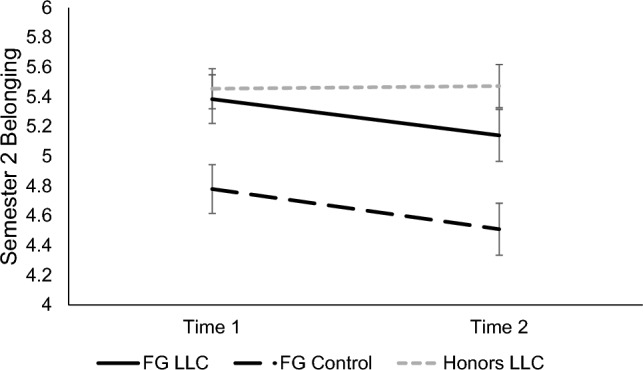
Participants’ feelings of belonging in biology in Semester 2. Means of participants’ feelings of belonging in their introductory biology course in Semester 2 for each timepoint are shown, separated by condition. Error bars signify ± 1 standard error.

Replicating Semester 1, a significant main effect of condition, *F*(2, 196) = 8.62, *p* < 0.001, *d* = 0.59, showed that overall, FG LLC students felt a stronger sense of belonging in biology (*M* = 5.26, *SE* = 0.13) than FG control group students (*M* = 4.64, *SE* = 0.16, *p* = 0.007, *d* = 0.31), but were statistically similar to honors LLC students (*M* = 5.46, *SE* = 0.13, *p* = 0.795). Students in the honors LLC also reported significantly stronger belonging in biology than those in the FG control group (*p* < 0.001, *d* = 0.41). A significant main effect of time, *F*(1, 196) = 5.68, *p* = 0.018, *d* = 0.33, revealed that students’ feelings of belonging decreased from Time 1 (*M* = 5.21, *SE* = 0.08) to Time 2 (*M* = 5.04, *SE* = 0.09) across condition. There was no significant time x condition interaction, *p* = 0.160. See Fig. 5.

#### Confidence

**Figure 6:**
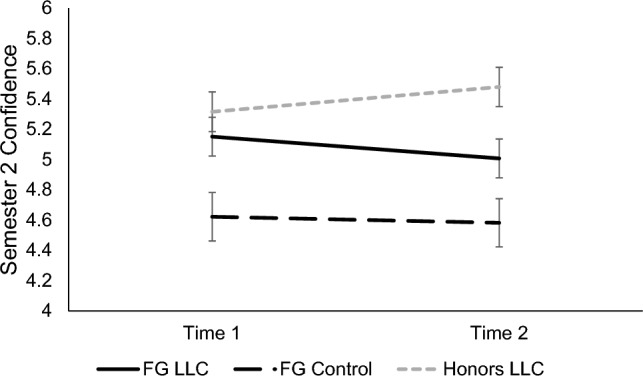
Participants’ feelings of confidence in biology in Semester 2. Means of participants’ feelings of confidence in their abilities in biology in Semester 2 for each timepoint are shown, separated by condition. Error bars signify ± 1 standard error.

A significant main effect of condition, *F*(2, 195) = 8.74, *p* < 0.001, *d* = 0.61, showed that FG LLC students (*M* = 5.08, *SE* = 0.12) reported more confidence in biology than FG control group students (*M* = 4.60, *SE* = 0.15; *p* = 0.037, *d* = 0.25). There was no difference in confidence between FG LLC versus honors LLC students (*M* = 5.40, *SE* = 0.12, *p* = 0.182). However, FG control group students reported significantly less confidence than honors LLC students (*p* < 0.001, *d* = 0.42). The effect of time, *p* = 0.909, and time x condition interaction were nonsignificant, *p* = 0.084. See Fig. 6.

#### Understanding the social relevance of biology

**Figure 7:**
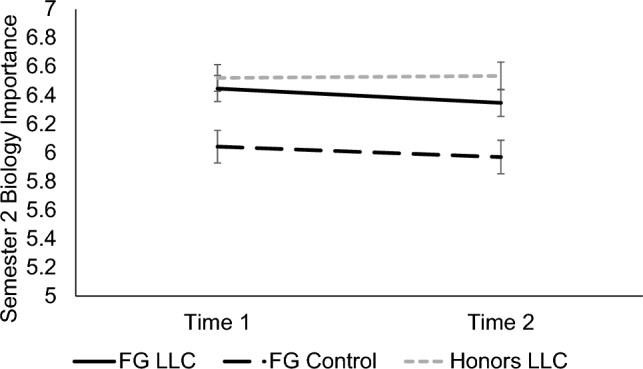
Participants’ perceived importance of biology in Semester 2. Means of participants’ perceived importance of biology in Semester 2 for each timepoint are shown, separated by condition. Error bars signify ± 1 standard error.

A significant main effect of condition, *F*(2, 191) = 8.00, *p* < 0.001, *d* = 0.58, showed that FG LLC students (*M* = 6.40, *SE* = 0.08) understood the connection between biological research and its relevance in everyday life more clearly than FG control group students (*M* = 6.01, *SE* = 0.10, *p* = 0.010, *d* = 0.30). Perceptions were statistically equivalent between the two LLC groups (honors LLC: *M* = 6.53, *SE* = 0.09, *p* = 0.800). Furthermore, students in the honors LLC felt that biology was more relevant to everyday life than FG control group students (*p* < 0.001, *d* = 0.39). Effects of time and time x condition interaction were nonsignificant, *p*s ≥ 0.320. See Fig. 7.

#### Anxiety

Unlike Semester 1, we did not find a significant main effect of condition, *F*(2, 196) = 2.07, *p* = 0.129. Instead, a significant main effect of time, *F*(1, 196) = 20.08, *p* < 0.001, *d* = 0.65, showed an overall increase in anxiety from Time 1 (*M* = 3.65, *SE* = 0.09) to Time 2 (*M* = 4.08, *SE* = 0.10), across conditions. A significant time x condition interaction, *F*(2, 196) = 9.60, *p* < 0.001, *d* = 0.62, showed that both the FG LLC group (Time 1: *M* = 3.27, *SE* = 0.15; Time 2: *M* = 4.24, *SE* = 0.17; *p* < 0.001, *d* = 0.44) and the honors LLC group (Time 1: *M* = 3.95, *SE* = 0.15; Time 2: *M* = 4.26, *SE* = 0.17; *p* = 0.045, *d* = 0.14) showed increased anxiety over time, while the control group did not (*p* > 0.99). In the discussion, we speculate how the COVID-19 pandemic may help explain this unexpected finding.

#### Motivation

We did not find a significant effect of condition on student motivation, *F*(2, 196) = 0.94, *p* = 0.394. A significant main effect of time, *F*(1, 195) = 20.60, *p* < 0.001, *d* = 0.64, showed that student motivation decreased from Time 1 (*M* = 5.68, *SE* = 0.06) to Time 2 (*M* = 5.34, *SE* = 0.09) across conditions. A significant time x condition interaction, *F*(2, 195) = 6.60, *p* = 0.002, *d* = 0.51, revealed that FG LLC showed less motivation over time (Time 1: *M* = 5.82, *SE* = 0.10; Time 2: *M* = 5.10, *SE* = 0.14, *p* < 0.001, *d* = 0.42), while participants in the control group and the honors LLC did not change over time (*p*s ≥ 0.149). Again, we discuss possible reasons for this unexpected finding in the discussion in the context of the COVID-19 pandemic.

#### Spring semester grades

Though we planned to compare final grades in the second semester of biology between the control and the FG LLC conditions, due to the unprecedented circumstances of the COVID-19 pandemic, students were allowed to change letter grading to pass/fail grading to preserve their cumulative grade point average. This led 32% of the sample to choose the pass/fail option, drastically reducing the analyzable sample of letter grades in the spring semester (control: *N* = 26, FG LLC: *N* = 44). Because the reduced sample of grades and the self-selection of students opting out of letter grades reduces the validity of grades, we did not analyze grades in the spring semester.

### Grades in other required STEM courses

We also examined whether the positive effects of the LLC would extend to students’ grades in other required STEM courses, such as calculus and general chemistry. We did not expect such an effect because the FG LLC was narrowly targeted to affect student experiences in biology by: (a) bringing a small cohort of FG students together in the same class; (b) brokering faculty mentoring relationships and exposing students to biology role models in class and first-year seminar; and (c) providing access to FG peer mentors in biology. Unlike the cohort-based introductory biology experience, FG students from the LLC condition and the control condition took calculus and chemistry in large lecture classes that were not for FG students only and not targeted to their needs.

We conducted *t*-tests comparing student grades in Calculus I, Calculus II, General Chemistry I, and General Chemistry II for the FG LLC group versus the FG control group during their first year of college. As we suspected, results revealed no significant differences in grades between the two FG conditions for all four classes in calculus and chemistry, *t*s ≤ 1.73, *p*s ≥ 0.089, suggesting that the positive effects of the FG LLC were specific to the biology learning environment and that the two groups of FG students (in the LLC versus the control condition) were academically similar, but for their biology LLC experience. See Supplementary Materials for separate results for each STEM course. The nonsignificant condition effects for calculus and chemistry grades compared to the significant condition effects for biology grades suggests that the “active ingredients” in the biology FG LLC may be its affinity-based cohort, small class size, access to role models and mentoring relationships with faculty and near-peers, or any combination of the above. The residential component may be less important given that the benefits of the LLC in this study accrued to students even in semesters when they were not living on campus during the pandemic.

### Persistence in biological science majors at the end of their second year

**Figure 8:**
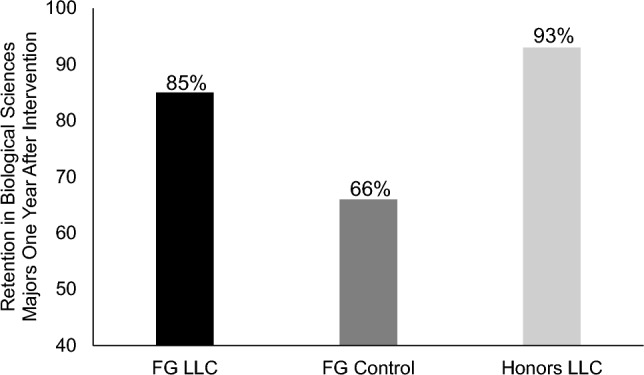
Percentage of participants retained in biological science majors one year after the end of the intervention. The average percentage of participants who were retained in their biological science majors at the end of their second year of college are shown, separated by condition.

At the conclusion of participants’ first year, the living learning community ended; FG students moved to other college residential halls where they were mixed with CG students and students across academic majors. We continued to track students’ persistence in biological science majors by accessing their college transcripts with their permission, through the end of their sophomore year. Our goal was to determine whether participation in an FG LLC in the first year of college would increase student retention in biological science majors one year after the intervention had ended relative to FG controls. For this analysis, we examined transcripts of students from both Semesters 1 and 2 who gave us permission to see their transcripts, resulting in a total sample of 307 students (control: *N* = 106, FG LLC: *N* = 75, honors LLC: *N* = 126). Student transcripts were coded; those who remained in a biological science major were coded as 1, while those who switched out of a biological science major were coded as 0. A significant chi-square test, χ^2^(2) = 28.62, *p* < 0.001, φ_c_ = 0.31, revealed significant differences across conditions. A larger percentage of students in the FG LLC (85%, 64 out of 75) remained biological science majors compared to the control condition (66%, 70 out of 106) where significantly more students switched majors (χ^2^(1) = 8.51, *p* = 0.001, φ_c_ = 0.22). Student retention in the biological sciences was statistically similar in the FG LLC versus honors LLC conditions (93%, 117 out of 126), (χ^2^(1) = 2.97, *p* = 0.085). Finally, retention was higher in the honors LLC compared to the FG control group (χ^2^(1) = 26.49, *p* < 0.001, φ_c_ = 0.34). Retention percentages are graphed in Fig. 8.

## Discussion

This present research is the first to investigate whether immersion in an LLC designed specifically for FG undergraduate students sparks and sustains their engagement in the biological sciences, through to the end of their second year of college, one year after the intervention had ended. We compared individuals who took part in a cohort-based program that combined intellectual and residential community with another group of students in the control condition who had a similar working-class FG history, similar academic interests, but were not in a working-class affinity cohort. The LLC directly addressed barriers faced by working-class FG students by creating an interdependent learning environment and providing them with resource-rich social networks through exposure to faculty and peer mentors who were FG juniors and seniors majoring in the biological sciences.

This study complements prior research in four ways. First, unlike previous work on FG students in STEM that used brief interventions to adjust students’ mindsets^18–21^, we developed an intervention that changed institutional features of learning environments to make them more welcoming, rather than expecting FG students to change their mindset to fit existing university structures. Second, unlike past research on LLCs that included a mixture of students from working-class, middle-class, and affluent backgrounds^25,26,29–31^, ours is one of the first studies to design and test the efficacy of an LLC dedicated to working-class students in STEM. Third, while most prior work on FG students and LLCs only measured course performance and/or persistence in STEM, we examined these metrics as well as an expansive list of psychological experiences in academic settings that are often precursors of students’ performance and decisions to persist in, or switch out of, STEM^35–38^. Finally, replicating and extending prior work on affinity-based STEM LLCs for other social groups (e.g., gender, race)^35,37,38^, we show that LLCs designed specifically for working-class students in biology also enhanced their grades and retention in biological science majors one year after the intervention.

In Semester 1, the first semester of college, immersion in an LLC with other FG peers significantly increased students’ feelings of belonging and motivation in biology, reduced anxiety, and improved end-of-semester grades in biology from an average of B to B+ compared to FG peers in the control group. The magnitude of these effects was statistically equivalent both before and during the COVID-19 pandemic, suggesting that the benefits of a learning community with other FG peers, rich network of mentoring relationships with faculty, advanced peers, and advisors, and smaller class sizes enhanced students’ academic experiences and grades, even without the residential component of the LLC. By the end of the second semester of college, we continued to find substantial benefits of LLC participation for FG students despite the significant disruption of residential life and teaching during the COVID-19 pandemic. Although cohort-based residential life was curtailed, first-generation students in the LLC continued to feel substantially more belonging in biology, more confidence, and believed biology to be more socially important in everyday life than the control group. A significantly larger percentage of FG LLC students persisted in biological science majors in their sophomore year compared to FG students in the control group.

Importantly, data from both semesters suggest that immersing FG students in an LLC closed the academic gap between them and honors students, who started college with substantial educational and economic privileges in terms of college guidance from family and high school preparation. For specificity, all analyses with the honors LLC condition (psychological experiences and retention) were also reconducted with only CG students in the honors college LLC. Results remained the same, regardless of whether the small number of FG students in the honors college LLC (*n* = 13) were included or excluded from the analyses. These findings suggest that immersion in an LLC reduces achievement gaps between FG and CG students in college.

Although overall, immersion in an identity-based LLC revealed clear benefits for FG students in both studies, a few inconsistencies emerged. First, the effect of the LLC on reducing student anxiety and increasing motivation was only found in Semester 1, not Semester 2. We speculate that this may have occurred because of the disruption caused by the COVID-19 pandemic during spring 2020 (Semester 2), as suggested by the overall increase in anxiety and decrease in motivation during that semester. At the start of the COVID-19 pandemic, students had to leave their on-campus residential learning community at short notice and their biology course migrated online to an asynchronous format. Given the pre-pandemic emphasis on communal living and in-person peer community in the LLC, its abrupt interruption likely contributed to elevated anxiety and reduced motivation especially in the group of first-generation students. A second inconsistent result was in Semester 2, where FG and honors LLC students reported significantly more confidence and a greater understanding of the social relevance of biology than FG control group students, though there were no group differences in Semester 1. Our speculation is that LLC students’ confidence about their ability in biology may have grown across the first year in college as they became more comfortable with college-level classes, hence the delayed effect. Similarly, weekly LLC seminars provided exposure to faculty research and social impact of that research, which may have increased students’ understanding of the social relevance of biology over time. One additional inconsistency between Semester 1 and 2 was that we did not test between-condition grade differences in biology in Semester 2. This is because during the COVID-19 pandemic students were given the option to convert their course grades to pass/fail grades. Many students chose this option in Semester 2; thus, we were unable to test for grade differences, unlike in Semester 1.

Our findings point to some important avenues of future research. First, our LLC intervention included several elements that need to be teased apart more carefully: small class size, learning with an affinity group of peers, living with these peers, access to role models, and mentoring relationships with faculty and near peers. Our data suggest that the residential component may be less important given that the benefits of the LLC accrued to FG students even in semesters when they were not living on campus during the pandemic. The absence of performance difference in math and chemistry classes between the LLC group versus the control group suggests that the two FG groups were otherwise academically similar and there was something special about the biology LLC environment responsible for the superior results, which could include any of the other LLC ingredients mentioned above. An important future direction would be to elucidate which elements of affinity-based programs are most impactful and which others are discretionary. Second, future work should also examine the generalizability of these findings to other marginalized identity groups in STEM (e.g., Black, Hispanic, and Indigenous students). Finally, it is also important to test the long-term effects of identity-based LLCs across the entire college experience beyond the first two years. We found that benefits of the one-year identity-based LLC program lingered through students’ first year and remained robust one year after the program had ended. It is unknown if these benefits endure even longer, spanning the entire college experience.

To truly promote diversity, equity, and inclusion in STEM, institutions need to take the responsibility to create welcoming learning environments that meet the needs of students who are traditionally marginalized in higher education and show that their success is valued, rather than expecting them to adjust on their own. Identity-based LLCs during the transition to college and other interventions that foster deeper relationships and communities of ingroup peers offer a secure psychological foundation for underrepresented students from which they can expand their sphere of exploration to find their place within the larger university.

## Method

### COVID disruption

The advent of the COVID-19 pandemic disrupted the experience of two student cohorts. The residential component of LLCs was disrupted for Cohort 2 (spring 2020) and Cohort 3 (fall 2020). Moreover, previously in-person biology courses were taught remotely in spring 2020 and academic year 2020–2021. Despite these disruptions, other features of the LLCs continued unabated, including small class sizes, weekly seminars, peer mentoring, and community-bonding activities, all live online on Zoom. The COVID pandemic provided us an unexpected opportunity to test if the affinity-based learning community with its enriched social relationships would continue to yield benefits, despite stripping out the residential in-person component.

### Semester 1: fall semester

In the first semester, we conducted a longitudinal quasi-experiment testing the impact of a non-traditional living-learning intellectual and social community of FG college students in biology set within a naturally existing university environment. Students were followed longitudinally from the middle to the end of the semester. Data were collected on their lived experience in academic settings and end-of-semester grades. This FG intervention group was compared to two comparison groups: FG students who were eligible for the LLC but were not in it, and honors students in an honors biology LLC who were primarily CG students.

#### Participants

Two hundred and forty-three students (*M*_*age*_ = 18.13 years, *SD* = 0.33) participated in our study during the fall semester of their first year in college. The sample comprised 162 females, 77 males, and 4 non-binary students. In terms of race/ethnicity, 42% were White, 22% Asian, 16% Black or African American, 10% Multiracial, 7% Hispanic, and 3% other ethnicities. All students were first-years, enrolled in the first introductory biology course required for all biological science majors. Data collection occurred during the fall semesters of 2018, 2019, and 2020. The intervention group (*n* = 86) included FG students in a biology LLC; the control group (*n* = 63) included first-generation students not in an LLC but who were eligible to participate; and the second comparison group (*n* = 94) included first-year students in the honors program who participated in a biology LLC for honors students only. Participants in the FG LLC condition completed the online survey while students were in their weekly seminar class, whereas students in the control and honors LLC groups were informed about the survey through class announcements and completed the online survey at a time that worked for them. Additionally, participants who completed the surveys were entered into a raffle for college memorabilia if they were in the 2018 cohort. Participants in the 2019 and 2020 cohorts received $10 for survey participation and were also entered into a raffle for one of three $100 cash prizes. Our study was approved by the University of Massachusetts Amherst Institutional Review Board. All study procedures were performed in accordance with ethical guidelines and informed consent was obtained from all participants.

#### Procedure

*Academic experience surveys*. All participants completed two surveys during the fall semester, once in the middle of the semester (4–6 weeks into the semester; Time 1) and again at the end of the semester (10–12 weeks into the semester; Time 2).

*Intervention group: first-generation LLC*. In the summer preceding their first year of college, all FG students planning to major in the biological sciences who had preregistered for introductory biology were invited to apply to take part in an LLC designed to be an affinity community of students from similar working-class backgrounds interested in biology. FG students who applied for the LLC were quasi-randomly assigned to the FG LLC or the FG control condition with one constraint. The LLC oversampled students from groups that are historically underserved in STEM: low-income students eligible for Pell grants (an indicator of family poverty), and students who identified as Black or Hispanic. These two groups were assigned into the FG LLC at a higher rate than the FG control condition (e.g., 41% of the intervention group were Black or Hispanic as compared to 26% of the control group).

There were 5 distinct features of the FG LLC. First, students took a lecture-style introductory biology course together (19–38 students). Second, students had a weekly seminar in which they heard research presentations by biological science faculty who were also FG and learned about research opportunities. Third, students had FG peer mentors who were sophomores or juniors in biology majors. Fourth, once a semester, LLC students gathered for community-building social activities. Fifth, students lived together in the same residential hall and had a roommate who was also in the FG LLC. While those in 2018 and 2019 (60% of the sample) experienced the residential component, those in the 2020 cohort were fully remote due to the COVID-19 pandemic and did not experience collective residence life (40% of the sample); this group also took their introductory biology course remotely, in a synchronous online format.

*FG control group*. Faculty instructors who taught introductory biology in the FG LLC also taught other sections of the same class that were open to all students. For our control group, we recruited FG students from these latter sections. All sections used the same syllabus, lectures, assignments, exams, and grading rubric; thus, the course content and instructors were identical across FG intervention and FG control conditions. However, students in the FG control group had a larger class size (350–400 students) than those the FG LLC group. Students in the FG control condition also attended a weekly first-year seminar (25 students per seminar) like the FG LLC group. However, unlike the FG LLC experience, classmates in the control group seminar were mostly CG students. Control group participants did not have an assigned FG peer mentor nor did they live with a roommate who shared their FG identity or academic interest in biology.

*Honors LLC*. Participants in the honors LLC were members of the honors college, who entered the university with high school records that exceeded their entering class average (i.e., approximately the top 13% of the incoming class). The vast majority of participants (90%) in the honors LLC were from college-educated families (continuing-generation or CG students). Like the FG LLC, students in the honors LLC took introductory biology as a cohort (43–48 students) and attended a weekly first-year seminar as a cohort, in which biological sciences faculty visited as guest speakers and presented their research. In 2018 and 2019, honors LLC students lived together in the same residence hall; their roommate was also in the same LLC (66% of the sample), while the 2020 cohort was remote during the pandemic (34% of the sample) and attended their introductory biology course live online.

#### Measures

*Survey measures*. At Time 1 (middle of the semester) and Time 2 (end of the semester), participants reported their lived experiences in their introductory biology class. All measures utilized a 1–7 Likert scale, from 1 (not at all) to 7 (very much).

*Belonging*. Four items were used to measure participants’ feelings of belonging in introductory biology adapted from prior research^43–46^. These items were: “I feel connected to my classmates in [course number],” “I feel accepted by my classmates in [course number],” “I feel like an outsider among my classmates in [course number] (reverse-coded),” and “I feel invisible in [course number] (reverse-coded).” These four items had robust reliability when averaged (Time 1: α = 0.81, Time 2: α = 0.83) to create a belonging index for each time point.

*Anxiety*. Four items were used to measure participants’ feelings of anxiety about their biology course (adapted from prior research^44,45,47–51^). These items were: “I feel worried about [course number],” “I feel stressed about [course number],” “I feel unsure about [course number],” and “[course number] is difficult this semester.” These four items (Time 1: α = 0.84, Time 2: α = 0.89) were averaged to create an anxiety index for each time point.

*Motivation.* Four items measured participants’ motivation to persist in introductory biology (adapted from prior research^44,45,47–51^). These items were: “I have the skills and abilities to successful in [course number] this semester,” “I will be able to overcome any challenges I experience in [course number] this semester,” “I have what it takes to handle [course number],” and “I feel confident about [course number].” These items were averaged to create a motivation index at each timepoint (Time 1: α = 0.93, Time 2: α = 0.94).

*Confidence*. Two items measured participants’ confidence in their ability in biology (adapted from prior research^41–43^). These items were: “In general, how confident do you feel about your ability in biology?” and “Do you think you have a knack or talent for biology?” These items were averaged to create an index of confidence at each timepoint (Time 1: α = 0.83, Time 2: α = 0.84).

*Social relevance of biology*. Two items measured the importance and relevance of biology in everyday life. These items were: “To what extent does biology solve important real-world problems that help people?” and “Is biology important in everyday life?” These items were averaged to create an index of biology relevance at each timepoint (Time 1: α = 0.69, Time 2: α = 0.73).

*Fall semester grades*. Participants’ written consent was requested to access their transcript from the university registrar. If permission was granted, we recorded students’ final letter grade in biology and converted it using the conventional 4.0 grading scale (i.e., A = 4.0, A− = 3.7, B+ = 3.3, B = 3.0 etc.). Students who took the class pass/fail were not included in these analyses. Because honors biology had slightly different content and a different team-based teaching method, honors course grades were not comparable to the typical biology course taken by FG students. Thus, grades in the honors LLC condition were not compared to the two FG groups, who took the same course with identical content and grading structure with the same instructor. We only compared grades between the FG LLC group and the FG control group.

### Semester 2: spring semester

Similar to Semester 1, we conducted a longitudinal quasi-experiment that tested the impact of an LLC for FG students. In Semester 2, we examined these effects during the second semester of introductory biology. Studies 1 and 2 had the same LLC participants (both FG and honors) but had different control group participants who were sampled from FG students enrolled in sections of introductory biology taught by the same instructor who also taught the FG LLC group.

#### Participants

One hundred and ninety-nine students (*M*_*age*_ = 18.36 years, *SD* = 0.51) participated in our study during the spring semester of their first year in college. The sample was comprised of 133 females, 62 males, and 4 nonbinary participants, and was 41% White, 26% Asian, 13% Black or African American, 10% Hispanic, 8% Multiracial, and 2.5% other ethnicities. All students were enrolled in their first year of college taking a second biology course that was part of the introductory sequence. Data collection occurred during spring 2019, 2020, and 2021. The intervention group (*n* = 76; 75 were the same as Semester 1) included FG students in a biological sciences LLC; the control group (*n* = 50; 10 students were the same as Semester 1 while 40 students were new) included FG students not in an LLC but who were eligible to participate; and the second comparison group (*n* = 73; 65 were the same as Semester 1) included first-year students in the honors program who were in a biological sciences LLC for honors students. Mirroring Semester 1, participants were entered into a raffle for college memorabilia for the 2019 cohort for completing the survey, while participants in the 2020 and 2021 cohorts received $10 and were also entered into a raffle for one of 3 $100 cash prizes.

#### Procedure

*Survey timeline*. Like Semester 1, all participants completed two surveys, once in the middle of the semester (4–6 weeks into the semester) and the other at the end of the semester (10–12 weeks into the semester). Participants in the control group and the honors LLC completed their survey outside of class time whereas those in the first-generation LLC completed their survey during their weekly seminar time.

*Intervention group: first-generation LLC*. All elements of the LLC were the same as Semester 1 (taking introductory biology and a weekly seminar together, peer advisors, community building activity, shared residential living). The FG LLC took the second biology class in the introductory biology sequence with the same instructor as students in the control group. The courses were identical in content and teaching style. Due to the COVID-19 pandemic, the residential component was disrupted for the 2020 cohort and the course migrated online (34% of the sample). The 2021 cohort (41% of the sample) lived on campus with other students in their LLC but took the biology class remotely due to the COVID-19 pandemic.

*FG control group*. Students in the control group were FG students taking the second biology class with the same instructor who also taught the FG LLC. The course content, teaching style, and evaluation were identical across both conditions. Like the previous study, this class had a larger class size (i.e., 210 to 350 students).

*Honors LLC*. Like the FG LLC, elements of the honors LLC were the same as Semester 1 (introductory biology class taught using a team-based learning format, a weekly seminar, and a residential component), and only enrolled honors LLC students. The 2020 cohort’s living community (38% of the sample) was partially disrupted; the 2021 cohort (34% of the sample) lived on campus with the fellow students in the LLC but took their introductory biology course in a live online format because of the pandemic.

#### Measures

Measures were identical to Semester 1. All measures had adequate to high reliability (belonging: αs ≥ 0.74, anxiety: αs ≥ 0.88, motivation: αs ≥ 0.89, confidence: αs ≥ 0.80, social relevance of biology: αs ≥ 0.80). We also recorded student grades by accessing their transcripts with their permission.

### Retention in biological science majors

One year after the LLC concluded, at the end of participants’ sophomore or second year in college, we examined their academic transcripts and recorded their major again. Those who remained in a biological science major (i.e., biology, microbiology, biochemistry, neuroscience, animal sciences, veterinary sciences) were coded as 1, while others who switched out of their biological science major were coded as 0.

### Math and chemistry grades

Participants’ written consent was requested to access their transcript from the university registrar. If permission was granted, we recorded students’ final letter grades in Calculus I, Calculus II, General Chemistry I, and General Chemistry II and converted it using the conventional 4.0 grading scale (i.e., A = 4.0, A− = 3.7, B+ = 3.3, B = 3.0 etc.). Students who took the class pass/fail were not included in these analyses.

### Supplementary Information

Supplementary Information.

## Data Availability

Participant data has been deposited in the Open Science Framework (OSF) repository and can be found at https://osf.io/pk3yz/?view_only=1655c1fb6ff44c8d9b36251c740705a9.

## References

[1] Fry R, Kennedy B, Funk C (2021). STEM Jobs see Uneven Progress in Increasing Gender, Racial and Ethnic Diversity.

[2] Georgeac OA, Rattan A (2022). Perceiving progress toward social equality: A model of signals and sense-making. Curr. Opin. Psychol..

[3] Starck JG, Sinclair S, Shelton JN (2021). How university diversity rationales inform student preferences and outcomes. Proc. Natl. Acad. Sci..

[4] American Physical Society. Building America’s STEM workforce: Eliminating barriers and unlocking advantages. In *American Physical Society Office of Government Affairs* (2021).

[5] Gibbons MM, Borders LD (2010). Prospective first-generation college students: A social-cognitive perspective. Career Dev. Q..

[6] Dennehy TC, Smith JS, Moore CD, Dasgupta N, Feldman R (2017). Stereotype threat and stereotype inoculation: Barriers and interventions that promote the success of underrepresented students in the first year of college. First Year Student Success.

[7] Thiem K, Dasgupta N (2022). From pre-college to career: Barriers facing historically marginalized students and research-driven solutions. Soc. Issues Policy Rev..

[8] Guryan J, Hurst E, Kearney M (2008). Parental education and parental time with children. J. Econ. Perspect..

[9] Ramey CT, Ramey SL, Kagan SL, Tarrant K (2010). The transition to school: Concepts, practices, and needed research. Transitions for Young Children: Creating Connections Across Early Childhood Systems.

[10] Bettencourt GM, Manly CA, Kimball E, Wells RS (2020). STEM degree completion and first-generation college students: A cumulative disadvantage approach to the outcomes gap. Rev. High. Educ..

[11] Phillips LT, Stephens NM, Townsend SSM, Goudeau S (2020). Access is not enough: Cultural mismatch persists to limit first-generation students’ opportunities for achievement throughout college. J. Personal. Soc. Psychol..

[12] Stephens NM, Fryberg SA, Markus HR, Johnson CS, Covarrubias R (2012). Unseen disadvantage: How American universities' focus on independence undermines the academic performance of first-generation college students. J. Personal. Soc. Psychol..

[13] Tate KA, Caperton W, Kaiser D, Pruitt NT, White H, Hall E (2015). An exploration of first-generation college students’ career development beliefs and experiences. J. Career Dev..

[14] Stephens NM, Townsend SS, Markus HR, Phillips LT (2012). A cultural mismatch: Independent cultural norms produce greater increases in cortisol and more negative emotions among first-generation college students. J. Exp. Soc. Psychol..

[15] Engle J, Bermeo A, O'Brien C (2006). Straight from the Source: What Works for First-Generation College Students.

[16] Laiduc G, Herrmann S, Covarrubias R (2021). Relatable role models: An online intervention highlighting first-generation faculty benefits first-generation students. J. First Gener. Stud. Success.

[17] Fernandez, M. J., Trenor, J. M., Zerda, K. S. & Cortes, C. First generation college students in engineering: A qualitative investigation of barriers to academic plans. In *2008 38th Annual Frontiers in Education Conference* T4D-1 (IEEE, 2008).

[18] Harackiewicz JM, Canning EA, Tibbetts Y, Giffen CJ, Blair SS, Rouse DI, Hyde JS (2014). Closing the social class achievement gap for first-generation students in undergraduate biology. J. Educ. Psychol..

[19] Harackiewicz JM, Canning EA, Tibbetts Y, Priniski SJ, Hyde JS (2016). Closing achievement gaps with a utility-value intervention: Disentangling race and social class. J. Personal. Soc. Psychol..

[20] Hecht CA, Priniski SJ, Tibbetts Y, Harackiewicz JM (2021). Affirming both independent and interdependent values improves achievement for all students and mitigates cultural mismatch for first-generation college students. J. Soc. Issues.

[21] Tibbetts Y, Harackiewicz JM, Canning EA, Boston JS, Priniski SJ, Hyde JS (2016). Affirming independence: Exploring mechanisms underlying a values affirmation intervention for first-generation students. J. Personal. Soc. Psychol..

[22] Dasgupta N (2011). Ingroup experts and peers as social vaccines who inoculate the self-concept: The stereotype inoculation model. Psychol. Inquiry.

[23] Dasgupta N, Scircle M, Hunsinger M (2015). Female peers in work teams enhance women’s motivation, verbal participation, and career aspirations in engineering. Proc. Natl. Acad. Sci..

[24] Eidum J, Lomicka L, Chiang W, Endick G, Stratton J (2020). Thriving in residential learning communities. Learn. Commun. Res. Pract..

[25] Sriram R, Diaz C (2016). STEM as "minority": A phenomenological case study of how students of color perceive their experience in a STEM living-learning program. J. Learn. Spaces.

[26] Inkelas KK, Vogt KE, Longerbeam SD, Owen J, Johnson D (2006). Measuring outcomes of living-learning programs: Examining college environments and student learning and development. J. Gener. Educ..

[27] Brower AM, Inkelas KK (2010). Living-learning programs: One high-impact educational practice we now know a lot about. Lib. Educ..

[28] Inkelas, K. K., Drechsler, M., Szelényi, K., Kim, Y. C., McCarron, G. P., Soldner, M., Brower, A. M., Crawford, S., Hempton, B. & Mainieri, T. (2007). National study of living-learning programs. In: *UW-Madison Customized Report*.

[29] Wawrzynski MR, Jessup-Anger JE (2010). From expectations to experiences: Using a structural typology to understand first-year student outcomes in academically based living-learning communities. J. Coll. Stud. Dev..

[30] Inkelas KK, Daver ZE, Vogt KE, Leonard JB (2007). Living-learning programs and first-generation college students’ academic and social transition to college. Res. High. Educ..

[31] Johnson DR, Soldner M, Leonard JB, Alvarez P, Inkelas KK, Rowan-Kenyon HT, Longerbeam SD (2007). Examining sense of belonging among first-year undergraduates from different racial/ethnic groups. J. Coll. Stud. Dev..

[32] Szelenyi K, Denson N, Inkelas KK (2013). Women in STEM majors and professional outcome expectations: The role of living-learning programs and other college environments. Res. High. Educ..

[33] Dagley M, Georgiopoulos M, Reece A, Young C (2016). Increasing retention and graduation rates through a STEM learning community. J. Coll. Stud. Retent. Res. Theory Pract..

[34] Reyes E, Neverett M, Farwell MA (2022). Supporting first generation student success in STEM through a living learning community. Underst. Intervent..

[35] Maltby JL, Brooks C, Horton M, Morgan H (2016). Long term benefits for women in a science, technology, engineering, and mathematics living-learning community. Learn. Commun. Res. Pract..

[36] Cline E, Bjӧrling E, Cilli-Turner E, Dinglasan-Panlilio J, Heller J, Kolodziej E, Kuo AC, Nahmani M, Sesko A, Wenderoth MP, Yeung KY (2021). Promoting academic success of economically disadvantaged, STEM-interested, first-and second-year undergraduate students via the ACCESS in STEM program at University of Washington Tacoma. Underst. Intervent..

[37] Domingo MRS, Sharp S, Freeman A, Freeman T, Harmon K, Wiggs M (2019). Replicating Meyerhoff for inclusive excellence in STEM. Science.

[38] Lee D, Harmon K (2013). The Meyerhoff scholars program: changing minds, transforming a campus. Metrop Univ..

[39] Ghazzawi D, Pattison D, Horn C (2021). Persistence of underrepresented minorities in STEM fields: Are summer bridge programs sufficient?. Front. Educ..

[40] Ashley M, Cooper KM, Cala JM, Brownell SE (2017). Building better bridges into STEM: A synthesis of 25 years of literature on STEM summer bridge programs. CBE Life Sci. Educ..

[41] Bradford BC, Beier ME, Oswald FL (2021). A meta-analysis of university STEM summer bridge program effectiveness. CBE Life Sci. Educ..

[42] Faul F, Erdfelder E, Buchner A, Lang AG (2009). Statistical power analyses using G* power 3.1: Tests for correlation and regression analyses. Behav. Res. Methods.

[43] Stout JG, Dasgupta N, Hunsinger M, McManus MA (2011). STEMing the tide: Using ingroup experts to inoculate women's self-concept in science, technology, engineering, and mathematics (STEM). J. Personal. Soc. Psychol..

[44] Dennehy TC, Dasgupta N (2017). Female peer mentors early in college increase women’s positive academic experiences and retention in engineering. Proc. Natl. Acad. Sci..

[45] Wu DJ, Thiem KC, Dasgupta N (2022). Female peer mentors early in college have lasting positive impacts on female engineering students that persist beyond graduation. Nat. Commun..

[46] Good C, Rattan A, Dweck CS (2012). Why do women opt out? Sense of belonging and women's representation in mathematics. J. Personal. Soc. Psychol..

[47] Jamieson JP, Mendes WB, Blackstock E, Schmader T (2010). Turning the knots in your stomach into bows: Reappraising arousal improves performance on the GRE. J. Exp. Soc. Psychol..

[48] Tomaka J, Blascovich J (1994). Effects of justice beliefs on cognitive appraisal of and subjective physiological, and behavioral responses to potential stress. J. Personal. Soc. Psychol..

[49] White JB (2008). Fail or flourish? Cognitive appraisal moderates the effect of solo status on performance. Personal. Soc. Psychol. Bull..

[50] Berry Mendes W, Gray HM, Mendoza-Denton R, Major B, Epel ES (2007). Why egalitarianism might be good for your health: Physiological thriving during stressful intergroup encounters. Psychol. Sci..

[51] Putwain DW, Symes W, Wilkinson HM (2017). Fear appeals, engagement, and examination performance: The role of challenge and threat appraisals. Br. J. Educ. Psychol..

