# Supplementary material for: An identity-based learning community intervention enhances the lived experience and success of first-generation college students in the biological sciences
Source: Sci Rep. 2024 May 3;14:10163. doi: 10.1038/s41598-024-60650-1 (PMC11068742; doi:10.1038/s41598-024-60650-1)
Supplement: Supplementary file 1 — Supplementary Information. [file 41598_2024_60650_MOESM1_ESM.docx]

**Supplementary Materials**

**Controlling for Demographic Differences Across Conditions**

Due to the nature of our living learning communities (i.e., a first-generation community and an honors community), as well as the oversampling of underrepresented racial minorities (i.e., Black and Latinx) into the FG LLC, we conducted one-way ANOVAs to test for condition differences in participants’ race and SAT scores. Follow-up post hoc tests were conducted using Bonferroni corrections to adjust for multiple comparisons. For race, we found that in both Semesters 1 and 2, significant main effects of race, *F*s ≥ 21.40, *p*s < .001, indicated that there was a higher proportion of Black and Latinx students in the FG LLC condition (55% in both semesters) than in the FG control condition (29% in Semester 1, 24% in Semester 2; *p*s < .001) and the honors LLC condition (13% in Semester 1, 10% in Semester 2; *p*s < .001). There were no racial composition differences between the control vs. honors LLC conditions, *p*s ≥ .076.

Significant main effects for SAT scores, *F*s ≥ 21.40, *p*s < .001, indicated that students in the honors LLC condition had higher average SAT scores (Semester 1: *M* = 1439; Semester 2: *M* = 1451) than both the FG LLC condition (Semester 1: *M* = 1218; Semester 2: *M* = 1216; *p*s < .001) and control condition (Semester 1: *M* = 1227; Semester 2: *M* = 1234; *p*s < .001). There were no differences in SAT scores between the two FG conditions, *p*s > .99.

We also conducted one-way ANOVAs on condition to test whether there were gender differences between conditions. There were also significant main effects of condition in both semesters, *F*s ≥ 3.05, *p*s ≤ .050, such that the FG LLC (Semester 1: 63% female, Semester 2: 62% female) had fewer female students than the control condition (Semester 1: 81% female, Semester 2: 84% female; *p*s ≤ .047). The proportion of female students in the honors LLC condition (Semester 1: 68% female, Semester 2: 71% female) did not differ from either FG condition, *p*s ≥ .214.

To ensure that the effect of the intervention (FG LLC, FG control, honors LLC) on student outcomes is not biased by demographic differences between conditions, we statistically controlled for student race, gender, and SAT scores using Analyses of Covariance (ANCOVA) when testing the effect of intervention conditions on each of the dependent variables. We controlled for student race (percentage of underrepresented racial minorities in each condition), student gender (percentage of female students in each condition), and students’ SAT scores. Mirroring our analysis strategy in the main text, for Semester 1, we conducted 3 Condition (control, FG LLC, honors LLC) x 2 Time x 2 Cohort (pre-pandemic, during pandemic) repeated measures ANCOVAs for each of the dependent measures. For Semester 2, we conducted 3 Condition (control, FG LLC, honors LLC) x 2 Time ANCOVAs for each of the dependent measures. We were not able to use the pre-pandemic vs. in-pandemic variable in the ANCOVAs for Semester 2 because the timing of the pandemic prevented us from having clean pre- and in-pandemic samples.

***Semester 1 Results While Controlling for Demographic Variables***

**Belonging.** We found a significant main effect of condition, *F*(2, 237) = 7.78, *p* < .001, *d* = 0.54, such that across the semester, FG students in the LLC (*M* = 5.61, *SE* = 0.15) felt a stronger sense of belonging in biology than FG students in the control group (*M* = 4.76, *SE* = 0.18, *p* < .001, *d* = 0.35). FG LLC students did not statistically differ from honors LLC students in belonging (*M* = 5.44, *SE* = 0.11, *p* > .99), but FG controls felt significantly less belonging in biology compared to the honors LLC (*p* < .001, *d* = 0.36). Those in the honors LLC (*M* = 5.34, *SE* = 0.15) did not significantly differ from either condition, *p*s ≥ .093. A significant main effect of cohort, *F*(1, 210) = 17.24, *p* < .001, *d* = 0.57, indicated that students reported more belonging in biology pre-pandemic (*M* = 5.57, *SE* = 0.09) than during the pandemic (*M* = 4.90, *SE* = 0.13). There were no significant main effect of time or interaction effects of time, condition, and cohort, *p*s ≥ .141).

**Anxiety.** Mirroring our results without covariates, in the ANCOVA with covariates, a significant main effect of condition, *F*(2, 237) = 9.46, *p* < .001, *d* = 0.60, revealed that FG students in the LLC reported less anxiety about biology class (*M* = 3.27, *SE* = 0.16) than FG students in the control group (*M* = 3.95, *SE* = 0.20, *p* = .015, *d* = 0.25) and students in the honors LLC (*M* = 4.36, *SE* = 0.17, *p* < .001, *d* = 0.44). There was no difference in anxiety between the FG control vs. honors LLC conditions (*p* = .478). Additionally, there were no significant main effects of time, cohort, or interaction effects involving time, condition, or cohort, *p*s ≥ .269.

**Motivation.** We found a significant main effect of condition for motivation, *F*(2, 210) = 5.17, *p* = .006, *d* = 0.44, such that FG LLC students reported significantly more motivation in biology (*M* = 5.89, *SE* = 0.14) than honors LLC students (*M* = 5.20, *SE* = 0.14, *p* = .008, *d* = 0.33). The FG control group did not significantly differ from either LLC group (*p*s ≥ .148). There was a significant interaction of time x cohort, *F* (1, 210) = 8.10, *p* = .005, *d* = 0.39, such that during the pandemic students’ motivation decreased from Time 1 (*M* = 5.59, *SE* = 0.14) to Time 2 (*M* = 5.35, *SE* = 0.13, *p* = .020, *d* = 0.32), whereas before the pandemic, students’ motivation did not significantly change over time, *p* = .115. There were no other significant effects, *p*s ≥ .070.

**Confidence.** Students’ confidence in biology did not vary significantly by condition or by time, *p*s ≥ .569. A significant time x condition interaction, *F*(2, 208) = 3.99, *p* = .020, *d* = 0.39, indicated that students in the FG control group showed a significant drop in confidence over time (*p* = .023, *d* = 0.32), whereas those in the FG and honors LLC did not significantly change in confidence over time (*p*s ≥ .140). A significant time x cohort interaction, *F*(1, 208) = 6.13, *p* = .014, *d* = 0.34, indicated that in pre-pandemic cohorts, students’ confidence marginally increased over time (*p* = .059), whereas during the pandemic, students’ confidence marginally decreased over time (*p* = .095). There were no other significant omnibus effects, *p*s ≥ .126.

**Understanding the Relevance of Biology in the Real World.** There were no significant effects of participants’ perceptions of the relevance of biology, *p*s ≥ .064.

***Semester 2 Results While Controlling for Demographic Variables***

As detailed in the main text, we did not examine the impact of cohort in our analyses for Semester 2 due to the timing of the pandemic, which prevented us from having clean pre- and during-pandemic subsamples. Thus, we present ANCOVAs in which we test the effects of time, condition, their interaction, and control for demographic variables.

**Belonging.** A significant main effect of condition, *F*(2, 171) = 4.09, *p* = .018, *d* = 0.44, showed that overall, FG LLC students felt a stronger sense of belonging in biology (*M* = 5.39, *SE* = 0.16) than FG control group students (*M* = 4.78, *SE* = 0.20, *p* = .026, *d* = 0.26), but no different from honors LLC students (*M* = 5.41, *SE* = 0.18, *p* > .99). Students in the honors LLC also did not significantly differ from the FG control group (*p* = .095). There were no other significant effects, *p*s ≥ .519.

**Confidence.** A significant main effect of condition, *F*(2, 170) = 4.31, *p* = .015, *d* = 0.45, showed that FG LLC students reported more confidence in biology (*M* = 5.22, *SE* = 0.14) than FG control group students (*M* = 4.66, *SE* = 0.17; *p* = .021, *d* = 0.27). There was no difference in confidence between FG LLC versus honors LLC students (*M* = 5.25, *SE* = 0.16, *p* > .99). The honors LLC students also did not significantly differ from the FG control group students (*p* = .078). There were no other significant effects, *p*s ≥ .153.

**Understanding the Social Relevance of Biology.** A significant main effect of condition, *F*(2, 191) = 8.09, *p* < .001, *d* = 0.62, showed that FG LLC students understood the connection between biological research and its relevance in everyday life more clearly (*M* = 6.45, *SE* = 0.10) than FG control group students (*M* = 5.89, *SE* = 0.13, *p* = .001, *d* = 0.27). Perceptions of relevance were statistically equivalent between the two LLC groups (honors LLC: *M* = 6.52, *SE* = 0.12, *p* > .99). Furthermore, students in the honors LLC felt that biology was more relevant to everyday life than FG control group students (*p* = .004, *d* = 0.30). There were no other significant effects, *p*s ≥ .697.

**Anxiety.** Unlike our analyses from the main text, we found a significant main effect of condition, *F*(2, 171) = 3.79, *p* = .025, *d* = 0.42, such that students in the FG LLC reported less anxiety (*M* = 3.54, *SE* = 0.18) than students in the honors LLC (*M* = 4.36, *SE* = 0.20; *p* = .025, *d* = 0.33). The FG control group fell in between (*M* = 3.60, *SE* = 0.22) and did not significantly differ from either group, *p*s ≥ .066. A significant time x condition interaction, *F*(1, 171) = 4.84, *p* = .009, *d* = 0.48, showed that both the FG LLC group (Time 1: *M* = 3.23, *SE* = 0.19; Time 2: *M* = 3.85, *SE* = 0.21; *p* < .001, *d* = 0.52) and the honors LLC group (Time 1: *M* = 4.04, *SE* = 0.21; Time 2: *M* = 4.67, *SE* = 0.23; *p* = .002, *d* = 0.47) increased in anxiety over time, while the control group did not (*p* = .506). There was no significant main effect of time, *p* = .115.

**Motivation.** There were no significant effects of time, condition, or time x condition interaction, *p*s ≥ .320.

***Summary***

In sum, after controlling for demographic variables of race, gender, and SAT scores, FG students in the LLC had a greater sense of belonging and reduced anxiety (Semester 1), as well as increased belonging, confidence, and understanding of the social relevance of biology (Semester 2), in comparison to FG students in the control group. As demonstrated by our analyses in Semester 1, these benefits were evident both before the pandemic and also during the pandemic, when the cohort-based residential life component of the LLC was disrupted but the cohort-based learning component was active.

**Fall Semester Grades by Cohort and Controlling for Covariates**

A 2 Condition (control vs. FG LLC) x 2 Cohort (pre-pandemic, during pandemic) ANOVA was conducted for fall semester grades. However, due to the small sample size during the pandemic (total: *N* = 33, control: *N* = 10, FG LLC: *N* = 23), we interpret these results with a great deal of caution. This analysis revealed a significant main effect of condition, *F*(1, 132) = 4.00, *p* = .047, *d* = 0.34, such students in the FG LLC condition earned higher grades (*M* = 3.35, *SE* = 0.08) than those in the control condition (*M* = 3.08, *SE* = 0.11). A significant main effect of cohort, *F*(1, 132) = 4.57, *p* = .034, *d* = 0.36, indicated that student grades were higher during the pandemic (*M* = 3.37, *SE* = 0.12) than before the pandemic (*M* = 3.07, *SE* = 0.06). This may have occurred because during the pandemic students were given special accommodation to opt to take required classes as pass/fail. Students who elected to retain a letter grade rather than reverting to pass/fail may have felt more confident about their performance in biology; thus, the pandemic effect on grades may be due to self-selection. Finally, there was no significant interaction of condition x cohort, *p* = .304.

In an ANCOVA in which we controlled for race, gender, and SAT scores, we also found a significant effect of condition, *F*(1, 122) = 10.15, *p* = .002, *d* = 0.58, such that those in the FG LLC condition earned higher grades (*M* = 3.40, *SE* = 0.07) than those in the control condition (*M* = 3.00, *SE* = 0.10). There were no significant effects of cohort of condition x cohort, *p*s ≥ .120.

In sum, student grades were significantly higher if they had participated in the FG LLC condition compared to the control condition, both before and during the pandemic, and even after controlling for demographic variations across conditions.

**Potential Limitations in Participant Selection**

As noted in Table S1, 38% of eligible students applied for the FG LLC. There are several reasons why more eligible students may not have applied: (1) some eligible students may not have been interested in residential living with only biology students; (2) some eligible students may have wanted to live in a particular part of campus or a particular residential hall that was different from where the FG LLC students were housed; (3) some eligible students may have missed the application deadline; and (4) others may have leaned "undecided" in terms of their major preference and may not have wanted to commit to a biology-focused LLC. These reasons are speculative and there may have been other reasons as well.

Moreover, as presented in Table S3, survey completion rates in the FG control and honors LLC conditions were lower than in the FG LLC condition, which likely occurred due to differences in the data collection method. As reported in the manuscript, in the FG LLC condition, we were able to administer surveys while students were in the LLC seminar class, which yielded a high response rate. However, for students in the control group and honors LLC group, we were only allowed to make an announcement in class about the option to participate in the survey; interested students signed up to take the survey outside of class time. Not surprisingly, the latter strategy reduced survey completion rates. It is also plausible that students in the FG control and honors LLC groups who chose to spend their free time to complete our surveys may have been more conscientious on average than students in the FG LLC or other students who did not take part in our surveys. Given that high conscientiousness is linked to better college outcomes and academic performance (e.g., Conrad & Patry, 2012; Richardson & Abraham, 2009), having such students in the control condition could have reduced mean differences between the FG LLC group versus FG control and honors LLC groups on our outcome variables than the actual true differences between these groups.

**References**

Conrad, N., & Patry, M. W. (2012). Conscientiousness and academic performance: A mediational analysis. *International Journal for the Scholarship of Teaching and Learning, 6*(1), n1.

Richardson, M., & Abraham, C. (2009). Conscientiousness and achievement motivation predict performance. *European Journal of Personality*, *23*(7), 589-605.

**Table S1.**

*Invitations to Apply, Applicants, and Admission into the First-Generation Biological Sciences Living Learning Community*

|  |  |  |  |  |  |
| --- | --- | --- | --- | --- | --- |
| Number | | |  | | |
| Students Invited to Apply | | | 466 | | |
| Students Who Applied to LLC | | | 133 | | |
| Students Admitted into the LLC | | | 93 | | |
| Students Who Accepted Offers | | | 91 | | |
|  | | |  | | |
| Demographics of Applicants | | | Accepted | Not Accepted |  |
| Gender | | |  |  |  |
| Female | | | 61/93 (66%) | 29/40 (73%) |  |
| Male | | | 32/93 (34%) | 12/40 (30%) |  |
| Race | | |  |  |  |
| Black or Hispanic | | | 51/93 (55%) | 12/40 (30%) |  |
| Other Ethnicity | | | 42/93 (45%) | 27/40 (68%) |  |
| Did Not Report | | | N/A | 1/40 (3%) |  |
| Pell Eligible | | |  |  |  |
| Yes | | | 52/93 (56%) | 16/40 (40%) |  |
| No | | | 41/93 (44%) | 23/40 (58%) |  |
| Did Not Report | | | N/A | 1/40 (3%) |  |

**Table S2.**

*Student Demographics Within Each Condition*

|  | | |  |  | | | |  | | | |
| --- | --- | --- | --- | --- | --- | --- | --- | --- | --- | --- | --- |
|  | FG Control | | | | | FG LLC | | | Honors LLC | | |
|  | Semester 1 | Semester 2 | | | Semester 1 | | Semester 2 | | | Semester 1 | Semester 2 |
| Gender |  |  | | |  | |  | | |  |  |
| Male | 12/63 (19%) | 9/50 (18%) | | | 32/86 (37%) | | 29/76 (38%) | | | 33/94 (35%) | 24/73 (33%) |
| Female | 50/63 (79%) | 40/50 (80%) | | | 54/86 (63%) | | 47/76 (62%) | | | 58/94 (62%) | 46/73 (63%) |
| Nonbinary | 1/63 (2%) | 1/50 (2%) | | | 0/86 (0%) | | 0/76 (0%) | | | 3/94 (3%) | 3/73 (4%) |
|  |  |  | | |  | |  | | |  |  |
| Race |  |  | | |  | |  | | |  |  |
| URM (% Black and Latinx) | 18/63 (29%) | 12/50 (24%) | | | 47/86 (55%) | | 42/76 (55%) | | | 12/94 (13%) | 7/73 (10%) |
|  |  |  | | |  | |  | | |  |  |
| White | 31/63 (49%) | 24/50 (48%) | | | 20/86 (23%) | | 16/76 (21%) | | | 52/94 (55%) | 41/73 (56%) |
| Black or African American | 13/63 (21%) | 3/50 (6%) | | | 24/86 (28%) | | 22/76 (29%) | | | 2/94 (2%) | 1/73 (1%) |
| Hispanic or Latinx | 3/63 (5%) | 7/50 (14%) | | | 10/86 (12%) | | 9/76 (12%) | | | 4/94 (4%) | 4/73 (6%) |
| Asian | 12/63 (19%) | 14/50 (28%) | | | 17/86 (20%) | | 16/76 (21%) | | | 24/94 (26%) | 21/73 (29%) |
| Multiracial | 1/63 (2%) | 2/50 (4%) | | | 13/86 (15%) | | 11/76 (15%) | | | 9/94 (10%) | 3/73 (4%) |
| Other Ethnicity | 3/63 (5%) | 0/50 (0%) | | | 2/86 (2%) | | 2/76 (3%) | | | 3/94 (3%) | 3/73 (4%) |
|  |  |  | | |  | |  | | |  |  |

**Table S3.**

*Survey Completion Rates*

|  | |  |  | |  | |
| --- | --- | --- | --- | --- | --- | --- |
|  | Time 1 | | | Time 2 | | Completed Both Timepoints |
|  |  | | |  | |  |
| Fall |  | | |  | |  |
| FG Control | 77/127 (61%) | | | 67/127 (53%) | | 63/127 (50%) |
| FG LLC | 89/92 (97%) | | | 87/92 (95%) | | 86/92 (93%) |
| Honors LLC | 115/134 (86%) | | | 97/134 (72%) | | 94/134 (70%) |
|  |  | | |  | |  |
| Spring |  | | |  | |  |
| FG Control | 57/85 (67%) | | | 52/85 (61%) | | 50/85 (59%) |
| FG LLC | 83/91 (91%) | | | 76/91 (84%) | | 76/91 (84%) |
| Honors LLC | 90/134 (67%) | | | 88/134 (66%) | | 73/134 (54%) |

**Table S4.**

*Grades in Other Required STEM Courses Separated by Condition*

|  |  | | | FG Control | FG LLC |  |
| --- | --- | --- | --- | --- | --- | --- |
| Course | | *N* | M(*SD*) | | M(*SD*) | *T*-test |
| Math | |  |  | |  |  |
| Calculus I | | 139 | 3.15 (*0.88*) | | 3.36 (*0.83*) | *t*(137) = 1.45, *p* = .151 |
| Calculus II | | 84 | 3.54 (*0.59*) | | 3.20 (*1.10*) | *t*(56.53) = 1.73, *p* = .089 |
|  | |  |  | |  |  |
| Chemistry | |  |  | |  |  |
| General Chemistry I | | 115 | 2.90 (*0.96*) | | 2.86 (*1.27*) | *t*(99.87) = 0.43, *p* = .853 |
| General Chemistry II | | 21 | 3.37 (*0.78*) | | 3.43 (*1.16*) | *t*(19) = 0.13, *p* = .898 |
